# Estimating nutrient uptake requirements for radish in China based on QUEFTS model

**DOI:** 10.1038/s41598-019-48149-6

**Published:** 2019-08-12

**Authors:** Jiajia Zhang, Ping He, Wencheng Ding, Xinpeng Xu, Sami Ullah, Tanveer Abbas, Chao Ai, Mingyue Li, Rongzong Cui, Chongwei Jin, Wei Zhou

**Affiliations:** 1 Ministry of Agriculture Key Laboratory of Plant Nutrition and Fertilizer, Institute of Agricultural Resources and Regional Planning, Chinese Academy of Agricultural Sciences (CAAS), Beijing, 100081 China; 2 Tianjin Institute of Agricultural Resources and Environment, Tianjin, 300192 China; 3 Institute of Agricultural Resources and Environment, Shandong Academy of Agricultural Sciences, Jinan, 250100 China; 4 The Ministerial Key Laboratory of Remediation of Pollution Environment and Eco-health, Zhejiang University, Hangzhou, 310029 China

**Keywords:** Ecology, Ecosystem ecology, Ecology

## Abstract

Imbalanced fertilization has caused lower yield and nutrient use efficiency for radish (*Raphanus sativus* L.) production in China. Estimating nutrient requirements for radish is crucial in optimizing fertilization to resolve the problem. On-farm experiments in the radish-growing regions of China from 2000 to 2017 were collected to investigate the relationship between fleshy root yield and nutrient accumulation in radish plant using the Quantitative Evaluation of the Fertility of Tropical Soils (QUEFTS) model. The QUEFTS model predicted a linear increase in fleshy root yield if nutrients were taken up in balanced amounts until yield reached about 60%–70% of the potential yield. The balanced N, P, and K requirements in radish plant simulated by the QUEFTS model were 2.15, 0.45, and 2.58 kg to produce 1000 kg of fleshy root, and the corresponding internal efficiencies (IEs, kg fleshy root per kg nutrient in total plant dry matter) for N, P, and K were 465.1, 2222.2, and 387.1 kg kg^−1^. The simulated balanced N, P, and K removal by fleshy root to produce 1000 kg fleshy root were 1.34, 0.30, and 1.93 kg, respectively. Approximately 62%, 67%, and 75% of N, P, and K in radish plant were presented in the fleshy root and removed from the soil. Field validation experiments confirmed the consistency between the observed and simulated nutrient uptake values. The QUEFTS model was proven to be effective for estimating nutrient requirements of radish and will contribute to develop fertilizer recommendations for radish cultivated in China.

## Introduction

Radish (*Raphanus sativus* L.) belongs to the family Brassicaceae and is an important vegetable worldwide because of its considerable adaptability and abundance of vitamins, soluble sugar, and folic acid, etc. In 2016, radish production area in China covered 1.2 million hectares and total production reached 44.6 million tons of fresh tuberous roots, which corresponded to 40% of the global land area used for radish production and 47% of the worldwide radish yield^1^, respectively. The appropriate fertilizer application is essential for ensuring high yield and excellent fleshy root quality of radish. However, radish growers generally over-apply chemical fertilizers, which caused nutrient supplies could not match up with crop nutrient requirement^2^. According to a fertilization survey, single-season nitrogen (N) fertilizer input for vegetables produced in southern and northern China reached more than 500 and 300 kg N ha^−1^, respectively, far exceeding crop requirements^3,4^. Excessive and unbalanced nutrient levels resulted in low nutrient use efficiency and unintended environmental consequences, as well as being detrimental to crop production and profitability^5–7^. Therefore, better nutrient management and fertilizer recommendations are expected to address these issues.

Some plant-based fertilizer recommendations, such as leaf color charts and chlorophyll meters have been applied for radish nutrient management^8^. However, many methods have been developed according to relatively little nutrient uptake data and are specific for individual nutrients without adequately consider the interactions among nutrients as the driving force for plant uptake. The balanced application of nutrients involves supplying specific nutrients based on plant requirements, which will maximize the efficiency of applied nutrients. Site-specific nutrient management (SSNM) is a dynamic management of nutrients to optimize the supply and demand of nutrients for a specific site or field, which was initially used for rice in the mid-1990s^9^. The use of the SSNM strategy reportedly increases yields and nutrient use efficiency in grain crops^10,11^ and enhances vegetable production^12^. However, there are many uncertainties regarding the requirements of N, P, and K for radish because the IE of radish varies widely depending on agronomic practices, nutrient supply, and the prevailing climate where the crop is cultivated, making it difficult to extrapolate the results from experimental stations to small fields^13^. Therefore, the SSNM strategy involves the use of quantitative and generic methods (e.g., simulation models) for estimating the relationship between yield and nutrient uptake, which will be relevant for making fertilizer recommendations^14^.

The Quantitative Evaluation of the Fertility of Tropical Soils (QUEFTS) model was selected for this study because it considers the interactions between N, P, and K, which is being the most prominent feature that distinguishes it from other models^15^. The QUEFTS model uses a large amount of nutrient uptake data, thus avoiding the problems related to using a few data for guiding fertilization. Additionally, it applies a function of line-parabolic-plateau to evaluate the relationship between crop yield and nutrient uptake and provides a generic method to estimate nutrient requirements for crops under a certain target yield, in view of the season-specific and climate-adjusted potential yield^16^. Thus, it represents a convenient tool for the application of the SSNM strategy^17–19^. The QUEFTS model was initially proposed for maize^20^, and has since been adapted for rice^19^, wheat^17,21^, and cassava^22^. However, it is never tried to estimate nutrient requirements for radish in vegetable systems. There is relatively little information regarding the balanced nutrition requirements of radish and the current study included considerable nutrient uptake data for radish produced in China. We hypothesized that the QUEFTS model can be used to estimate the requirements of nutrient and contribute to make fertilizer recommendations for radish in China. Given that, the objectives of the present investigation of radish nutrient requirements were as follows: (1) determine the envelope functions describing the relationships between fleshy root yield of radish and nutrient uptake across diverse growing environments in China; (2) estimate the balanced requirements of N, P, and K for radish using the QUEFTS model; and (3) evaluate the N, P, and K uptake of radish simulated by the QUEFTS model through field experiments.

## Materials and Methods

### Data source

In China, radish crops are classified according to the growing season (i.e., spring radish, summer radish, autumn radish, and winter radish), with autumn radish dominating in most regions. Moreover, the land on which radish is cultivated corresponds to about 20%–50% of the area used to produce autumn vegetables, which is an important source of vegetables^23^. Radish plants are cultivated over a relatively short period for their tuberous roots, which assume diverse shapes (round, oval, or elongated), sizes, and colors (red, green, or white), and are associated with various flavors. In addition to monocropping cultivation practices, radish cropping systems mainly include broccoli (*Brassica oleracea*)–spring radish, potato (*Solanum tuberosum* L.)–autumn radish, cabbage–autumn radish, and tomato (*Lycopersicon esculentum* Mill.)–autumn radish. Due to differences in climates, radish sowing and harvesting dates vary widely across different regions in China.

**Figure 1:**
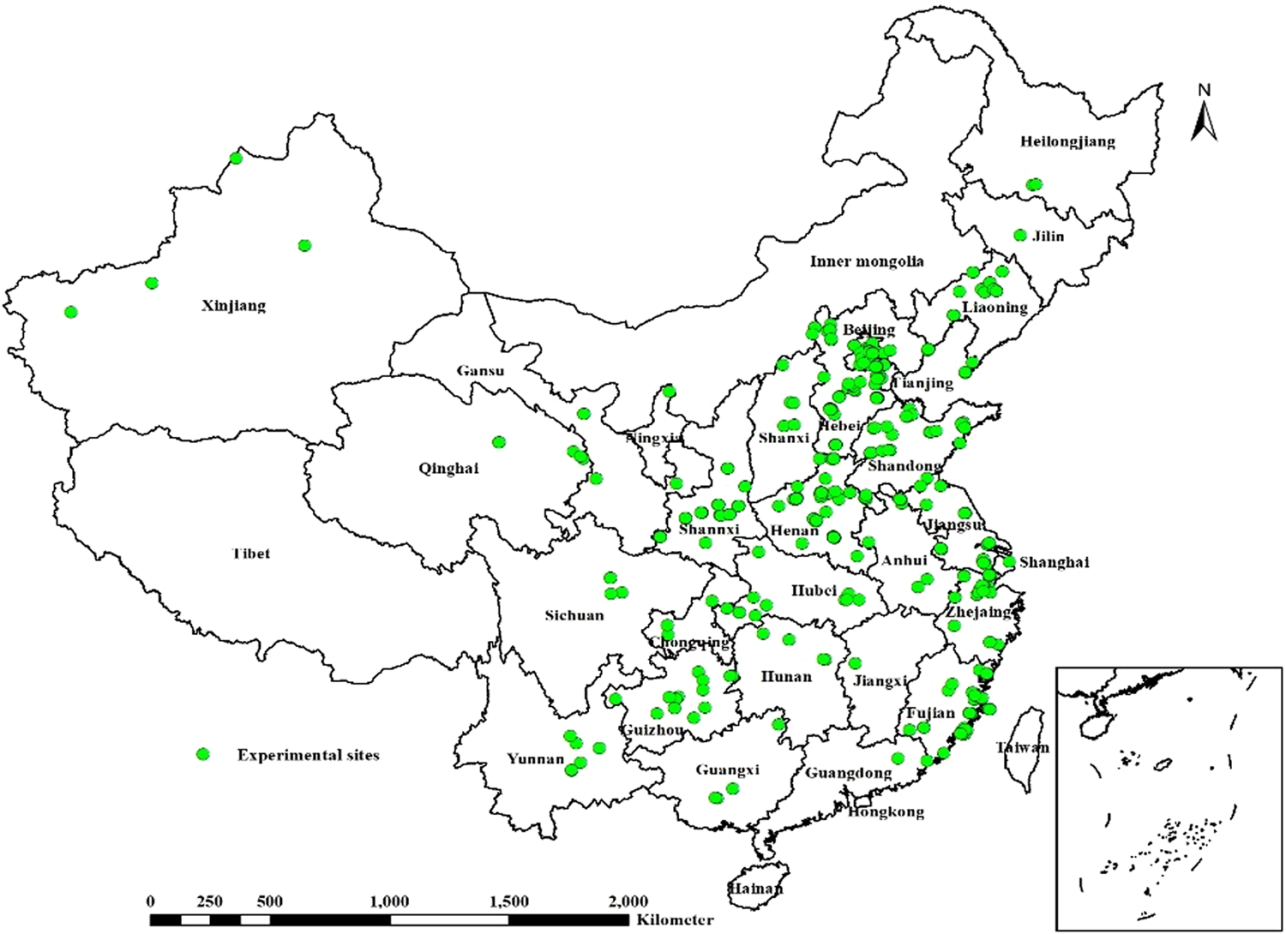
Geographical distribution of experimental sites for radish cultivation in China (2000–2017).

Radish datasets for the fleshy root yield; N, P, and K uptake by the fleshy roots and leaves; harvest index (HI, kg fleshy root dry matter per kg plant dry matter); and the amount of fertilizer application were established. The radish datasets were compiled from unpublished various field experiments studies conducted by the International Plant Nutrition Institute (IPNI) China Program and our group and published articles (2000–2017) available in the China Knowledge Resource Integrated Database^24^ regarding 28 provinces or municipalities in China (Fig. 1). As the radish datasets keep updating over time and needs to include all available latest data, the data from field validation experiments between 2016 and 2017 described in the following ‘Model validation’ section was also supplemented to the latest datasets to provide a more representative dataset. Field experiments examined the effects of diverse cultivation practices, farmers’ practice, optimal nutrient management, nutrient omission treatments, and different fertilizer application rates across radish-growing regions in China. Consequently, the field experiments involved various soils, climatic conditions, and agrotechniques (Table 1). Cultivation practices, including irrigation and the management of diseases, pests, and weeds, were determined by local researchers to ensure high yields.

**Table 1:** Characteristics of the experimental sites and the soil properties in the radish-producing regions of China.

Province	Climate type	Radish season	Case (n)	Latitude (°N)	Longitude (°E)	pH	Organic matter (%)	Alkali-hydrolysable N (mg kg^−1^)	Olsen P (mg kg^−1^)	NH_4_OAc-K (mg kg^−1^)
Jilin	Cool temperate	Autumn	5	43.73–43.82	125.09–125.37	6.5	1.8	50.2	25.5	173
Liaoning		Autumn	43	38.84–42.71	121.17–124.04	5.4–7.8	1.2–3.3	32.5–65.6	20.5–67.0	75.0–290.1
Heilongjiang		Autumn	15	45.68–45.70	126.62–126.77	6.6–7.3	1.9–2.6	50.2–86.7	27.8–85.6	120.5–360.4
Shannxi	Temperate	Summer and Autumn	82	33.06–35.99	106.16–110.15	7.1–8.6	0.9–1.7	36.5–115.5	5.9–60.4	93.0–191.0
Ningxia		Autumn	4	39.08	106.67	7.6	1.1	51.0	10.5	145
Gansu		Autumn	34	35.44–38.18	102.50–106.95	7.7–8.8	1.3–1.9	48.2–234.2	16.3–37.6	130.3–233.9
Xinjiang		Autumn	16	39.25–46.72	76.80–87.56	7.6–8.1	0.6–0.9	55.2–80.4	3.9–5.7	220.5–289.0
Qinghai		Autumn	70	36.41–36.93	98.48–102.54	7.9–8.2	1.1–1.5	17.3–89.0	30.4–62.0	88.4–184.0
Beijing	Temperate	Autumn	119	34.65–40.46	112.50–117.09	7.1–7.8	1.0–1.9	81.0–105.0	27.5–86.6	112.0–177.0
Tianjin		Spring and Autumn	263	34.48–40.12	112.59–117.77	7.9–8.4	1.4–4.5	115.4–207.8	18.4–173.2	264.8–365.0
Shanxi		Autumn	12	37.52–39.95	112.17–112.67	7.4–8.1	1.1–1.8	46.5–88.3	15.1–17.5	85.0–200.1
Shandong		Spring and Autumn	226	34.65–37.13	115.00–120.76	5.2–8.2	0.5–2.5	18.0–78.0	5.7–89.0	28.3–196.0
Henan		Autumn	274	32.16–40.49	111.65–116.94	6.2–8.0	0.1–4.9	52.6–117.0	12.5–79.6	28.4–210.0
Hebei		Spring and Autumn	312	36.13–41.39	113.75–119.60	7.3–9.0	0.4–3.1	32.7–171.4	2.8–28.8	49.2–184.5
Anhui	Temperate sub-tropical	Autumn and Winter	31	30.69–34.06	115.59–117.98	6.1–8.1	2.4–3.3	105–173.5	23.7–184.6	157.2–212.0
Hubei		Autumn	83	30.11–32.61	109.04–114.90	4.5–8.4	1.2–4.0	45.6–145.0	7.6–38.8	75.0–191.6
Hunan		Autumn	45	28.15–29.37	110.58–113.17	4.5–6.0	1.6–2.3	101.0–110.0	19.2–38.0	120.0–276.0
Jiangsu		Autumn	60	31.22–34.55	117.24–120.89	7.2–8.5	2.2–3.4	40.3–195.8	13.4–16.4	79.8–95.9
Jiangxi		Autumn	3	27.92	114.44	5.5	2.2	80.4	40.4	90.0
Shanghai		Autumn	5	31.19	121.73	6.5	0.3	209.0	167.0	178.0
Zhejiang		Spring and Autumn	145	27.98–30.78	118.88–122.53	5.1–7.9	0.6–5.3	47.8–276.9	4.5–128.0	33.1–282.5
Chongqing	Sub-tropical	Autumn	23	29.44–30.76	106.42–108.39	6.9–7.7	0.5–1.7	58.0–106.0	20.5–128.0	35.0–112.0
Guizhou		Summer and Autumn	106	26.10–27.93	104.17–109.16	5.6–7.7	0.8–3.7	58.3–143.0	11.0–36.3	51.0–220.0
Yunnan		Autumn	38	23.95–25.35	102.30–103.51	4.9–6.6	0.6–2.5	60.6–184.3	5.2–58.9	39.0–280.5
Sichuan		Autumn	31	31.09–31.70	103.90–104.42	6.8–7.9	0.8–5.1	42.9–133.3	19.7–20.5	56.4–125.0
Guangxi	Tropical	Autumn	38	22.81–25.70	108.25–111.01	4.9–6.5	2.2–3.2	89.5–143.6	27.4–54.1	38.0–263.2
Guangdong		Autumn	19	23.71–23.94	115.78–116.94	4.5–6.6	1.8–5.3	42.9–115.1	10.6–65.6	32.5–170.9
Fujian		Spring and Autumn	186	23.96–30.36	116.41–120.46	5.0–7.0	0.6–3.3	57.0–174.7	2.9–70.6	35.0–352.5

### Development of QUEFTS model

The QUEFTS model was originally proposed by Janssen *et al*. (1990), assuming that internal efficiency (IE) is constant until the yield reaches about 70%–80% of the potential yield^15,25^. The IE is used to assess the ability of plants to convert nutrients obtained from various sources into economic yields^26^. In the model, the 2.5th, 5.0th, and 7.5th percentiles of all calculated IE data were used as the maximum accumulation (a) and the maximum dilution (d) of nutrient levels to define the envelope functions. Data with harvest indexes (HIs) below 0.4 were eliminated because they were assumed to indicate the crop was exposed to other abiotic or biotic stresses due to factors other than the nutrient supply^27^. The resulting dataset for plants whose growth wasn’t restricted by factors other than N, P, and K was required to calibrate the QUEFTS model. The steps of the QUEFTS model have been described in the previous studies^14,17,18^. Additionally, the nutrient uptake under different potential yields and targets yields was estimated using the solver model in Microsoft Office Excel. The key steps were as follows: (a) selecting appropriate data to fulfil the model boundary conditions; (b) defining the two borderlines of N, P, and K corresponding to the maximum and minimum nutrients accumulation in plant; and (c) simulating the optimal N, P, and K uptake curves under different target yields or potential yields.

### Model validation

The QUEFTS model was validated on 63 farm fields at the following locations: Tianjin (21 fields) and Shandong (18 fields) (from autumn 2016 to spring 2018), Hebei (9 fields) and Zhejiang (6 fields) (from autumn 2016 to autumn 2017), and Beijing (9 fields) (spring 2018). Radish is a popular local vegetable that is typically cultivated in open fields in spring and autumn at the selected locations. At all sites, spring radish was grown from early or mid-April to mid- or late June, while autumn radish was sown in early or mid-August and harvested in late October or early November. The nutrient recommendations were from the Nutrient Expert (NE) decision support system for radish^28^. The NE system was developed by IPNI and adopts the SSNM principles and the QUEFTS model (based on radish datasets from 2000 to 2015) to simulate the optimum nutrient requirements for crops^28,29^. The target agronomic efficiency of applied N and the expected yield response are the bases for determining N requirements, whereas for P and K fertilizer recommendations, internal nutrient efficiency combined with estimations of attainable yield, balance, and yield response are all important in the NE system^28^. The P and K balances are calculated by considering the residual effects of previously applied fertilizers as well as crop residues to avoid excessive addition of nutrients in the soil. The yield response to N, P, or K is the yield difference between plots with sufficient N, P, and K nutrients and those lacking one of these nutrients. The agronomic efficiencies of N, P, and K represent the yield increase when one unit of N, P_2_O_5_, or K_2_O applied. On the basis of the NE fertilizer recommendation, the rates of N, P and K fertilizers application ranged from 145–170 kg N ha^−1^, 62–119 kg P_2_O_5_ ha^−1^, and 160–278 kg K_2_O ha^−1^, respectively. Specifically, N was applied as urea (46% N), while P and K were applied as a single superphosphate (12% P_2_O_5_) and potassium sulfate (50% K_2_O), respectively. The spring radish and autumn radish varieties used in this study were all commonly grown at the selected locations because of their high yields.

For all experiments, the plots were 20–30 m^2^ and included five rows with the following plant densities (line spacing and plant spacing): 50 × 30 cm in Tianjin, Hebei, and Beijing; 65 × 20 cm in Shandong; and 15 × 15 cm in Zhejiang. The N and K fertilizers were applied as a basal fertilizer, a topdressing made by hole application between plants at the rosette stage, and at the fleshy root expanding stage. The P fertilizer and the basal N and K fertilizers were incorporated into the soil before sowing. Additionally, agronomic practices including irrigation, insect and weed control, tillage, etc, were completed according to the optimum local management strategies. Disease symptoms were not observed throughout the study.

At harvest, plant samples were collected from the middle three rows in each plot. Plants were harvested manually, after which the fleshy roots and leaves were weighed (i.e., fresh weight) separately. The harvested plant materials were then oven-dried at 70 °C to determine the dry weight. Subsamples of fleshy roots and leaves were collected to determine the concentrations of N, P, and K. The fleshy roots and leaves were digested with H_2_SO_4_–H_2_O_2_, and the concentrations of total N, P, and K were determined adopting the Kjeldahl approach, vanado-molybdate yellow color approach, and flame spectrophotometers, respectively. The accumulation of N, P, and K in plant was calculated by multiplying the plant dry weight by the nutrient concentration. Additionally, the nutrient uptake was applied to analyze the correlation between the QUEFTS model simulated and observed nutrient uptake.

The root mean square error (RMSE), normalized RMSE (nRMSE), and mean error (ME) equations provided below were used to evaluate the QUEFTS model and the deviation between measured and simulated data.

1 $${\rm{RMSE}}=\sqrt{\frac{{\sum }_{i=1}^{n}{({s}_{i}-{m}_{i})}^{2}}{n}}$$

2 $${\rm{nRMSE}}=\frac{{\rm{RMSE}}}{\bar{m}}$$

3 $${\rm{ME}}=\frac{{\sum }_{i=1}^{n}({s}_{i}-{m}_{i})}{n}$$

Where *s*_*i*_ and *m*_*i*_ represented the values of simulated and measured nutrient uptake (kg ha^−1^), respectively; *n* was the number of values; and $\bar{m}$ represented the average value of measured nutrient uptake (kg ha^−1^). The equations of RMSE and ME measured the average discrepancy between the simulated and measured data with the same unit (kg ha^−1^), while the nRMSE equation did not consider the unit and enabled comparisons among values with different units.

### Statistical analysis

The SAS 9.3 software (SAS Institute, Inc., Cary, NC, USA) was used to analyze the significance of any differences between the average values of simulated and measured nutrient uptake based on Student’s *t*-test at the 0.05 significance level.

## Results and Discussion

### Fleshy root yield and nutrient uptake

The average fleshy root yield of radish was 63.5 t ha^−1^, ranging from 4.6 to 119.8 t ha^−1^ from field experiments in the radish database during the 2000–2017 periods (Table 2). The variability in the radish yield was due to different radish varieties, climates, and agronomic practices^30–32^. The average radish yield in this study was greater than the 35.0 t ha^−1^ reported in the China Agriculture Statistical Report (2017)^33^, likely because of the differences in radish varieties and nutrient management practices between the field experiments in the radish database and farmers’ fields. The average value of HI was 0.64 kg kg^−1^, ranging from 0.34 to 0.91 kg kg^−1^.

**Table 2:** Radish characteristics, including fleshy root and leaf yield (fresh weight), total dry matter (DM), harvest index, N, P, and K accumulation in fleshy root, leaf, and total plant DM, concentrations of N [N], P [P], and K [K] in fleshy root and leaf, and nutrient harvest index (kg nutrient in fleshy root per kg nutrient in total plant DM).

Parameter	Unit	Case (n)	Mean	SD^a^	Minimum	25%Q^b^	Median	75% Q	Maximun
Fleshy root yield	t ha^−1^	2288	63.5	26.4	4.6	44.2	63.0	83.0	119.8
Leaf yield	t ha^−1^	675	25.1	16.1	0.5	12.7	22.1	31.7	90.0
Total dry matter	t ha^−1^	515	5.4	2.2	0.4	3.7	5.4	7.0	10.5
harvest index	kg kg^−1^	515	0.64	0.10	0.34	0.57	0.64	0.69	0.91
N in fleshy root	kg ha^−1^	546	92.2	41.0	4.0	61.2	85.7	121.1	217.3
P in fleshy root	kg ha^−1^	541	19.9	9.7	1.1	13.7	18.3	24.8	68.3
K in fleshy root	kg ha^−1^	545	150.4	69.7	8.8	106.2	140.2	196.6	357.7
N in leaf	kg ha^−1^	524	63.9	34.2	4.9	34.7	59.9	84.6	185.0
P in leaf	kg ha^−1^	519	10.4	8.6	0.6	4.7	7.9	12.5	49.4
K in leaf	kg ha^−1^	523	65.4	50.5	3.8	24.4	54.7	84.8	232.3
[N] in fleshy root	g kg^−1^	546	28.0	7.0	7.5	24.0	28.5	32.7	47.1
[P] in fleshy root	g kg^−1^	541	6.2	2.5	1.7	4.3	6.1	7.7	18.2
[K] in fleshy root	g kg^−1^	545	46.7	18.1	10.7	35.3	45.4	56.2	102.1
[N] in leaf	g kg^−1^	534	32.7	6.3	9.4	29.0	32.4	36.3	54.4
[P] in leaf	g kg^−1^	529	5.1	2.6	0.5	3.5	4.3	5.9	18.1
[K] in leaf	g kg^−1^	533	31.7	14.1	5.2	21.5	32.0	42.0	78.2
N in total DM	kg ha^−1^	539	155.4	64.8	11.3	102.8	151.6	206.7	333.2
P in total DM	kg ha^−1^	529	30.9	13.9	1.7	20.6	29.7	38.9	78.5
K in total DM	kg ha^−1^	533	217.9	110.3	14.4	133.6	205.0	278.4	517.4
N harvest index	kg kg^−1^	536	0.60	0.12	0.13	0.52	0.61	0.68	0.91
P harvest index	kg kg^−1^	531	0.68	0.14	0.21	0.61	0.71	0.78	0.96
K harvest index	kg kg^−1^	535	0.72	0.12	0.23	0.63	0.73	0.82	0.94

^a^SD, standard deviation.

^b^Q, quartile.

The average concentrations of N, P, and K in fleshy roots were 28.0, 6.2, and 46.7 g kg^−1^, respectively, while the concentrations of N, P, and K in leaves were 32.7, 5.1, and 31.7 g kg^−1^, respectively. The average values of plant N, P, and K uptake were 155.4, 30.9, and 217.9 kg ha^−1^, respectively. However, there were considerable variations in the nutrient concentrations in the fleshy roots (7.5–47.1 g N kg^−1^, 1.7–18.2 g P kg^−1^, and 10.7–102.1 g K kg^−1^) and leaves (9.4–54.4 g N kg^−1^, 0.5–18.1 g P kg^−1^, and 5.2–78.2 g K kg^−1^). This variability reflected the diversity in the environmental conditions and nutrient availability. The lower nutrient concentrations were mainly derived from nutrient-deficiency plots with optimal growth conditions. The higher nutrient concentrations were obtained from excessive N, P, and K application, while environmental conditions or other nutrients strongly limited the crop growth. The nutrient HIs of N, P, and K (kg nutrient in fleshy root per kg nutrient in total plant dry matter) were 0.60, 0.68, and 0.72 kg kg^−1^, respectively, meaning that about 60%, 68%, and 72% of the N, P, and K in plants, respectively, were in the fleshy root. The amount of P and K in the fleshy root harvested from the field was used to assess the replacement requirements of P and K fertilizers to achieve a target yield and sustain P and K in the soil. The diversity in how experiments were conducted (i.e., different sites, seasons, and management practices), likely explained the potential variation in radish characteristic parameters including yield, nutrient uptake, nutrient concentration, and harvest index.

### Internal efficiency and reciprocal internal efficiency

The relationship between fleshy root yield and N, P, and K uptake in radish plant was estimated using the IE and reciprocal internal efficiency (RIE, nutrient in total plant dry matter per 1000 kg fleshy root). In radish database corresponding to several treatments, the average IE values of N, P, and K were 455.0, 2327.3, and 361.9 kg fleshy root (fresh matter) kg^−1^ (Table 3), respectively, ranging from 182.1 to 1213.0 kg kg^−1^, 757.4 to 6118.9 kg kg^−1^, and 132.5 to 1043.4 kg kg^−1^. To produce 1000 kg fleshy root, the average N, P, and K requirements were 2.45, 0.49, and 3.41 kg, respectively, ranging from 0.82 to 5.49 kg for N, 0.16 to 1.32 kg for P, and 0.96 to 7.55 kg for K. The IE-N and IE-K were greatly affected by nutrient management. Because of the limited availability of nutrients, N and K concentrations were more diluted in plants grown in the N and K omission plots, respectively, than in plants treated with fertilizers. Thus, the IEs were greater for plants grown in the omission plots than for plants grown in plots with ample nutrients. The large variations in IEs observed from field experiments in radish database reflect the season- and site-specific differences in environmental conditions (e.g., temperature) as well as the nutrient unbalances and problems with irrigation and weed and pest control. Thus, a modeling method that can evaluate the ‘true’ optimal nutrient requirements should be developed.

**Table 3:** Descriptive statistics of the internal efficiency of N, P, and K (IE, kg fleshy root per kg nutrient) and its reciprocal internal efficiency (RIE, kg nutrient per 1000 kg fleshy root) for radish grown in China.

Parameter	Unit	Case (n)	Mean	SD^a^	Minimum	25%Q^b^	Median	75%Q	Maximum
IE-N	kg kg^−1^	539	455.0	157.7	182.1	349.8	422.5	530.0	1213.0
IE-P	kg kg^−1^	529	2327.3	859.5	757.4	1770.3	2133.1	2807.3	6118.9
IE-K	kg kg^−1^	533	361.9	196.8	132.5	229.3	284.1	405.1	1043.4
RIE-N	kg t^−1^	539	2.45	0.79	0.82	1.89	2.37	2.87	5.49
RIE-P	kg t^−1^	529	0.49	0.18	0.16	0.36	0.47	0.56	1.32
RIE-K	kg t^−1^	533	3.41	1.33	0.96	2.47	3.52	4.36	7.55

^a^SD, standard deviation.

^b^Q, quartile.

### Selecting data to adapt the QUEFTS model

Determining the maximum accumulation (a) and maximum dilution (d) boundary lines using the QUEFTS model required a data set in which plant growth was not limited by factors other than the supply of N, P, and K. However, it is likely that this criterion was not met for all field experiments in the radish database. Prior to calibrating the QUEFTS model, we excluded data points that represented plants with adequate nutrient uptake to maintain greater yield but suffered with drought, pests, and disease stresses. Previous studies on grain crops and cassava showed that the HI could be used as an effective tool to select data for calibrating the model^14,27^, and data with HI below 0.4 was eliminated because lower HI indicated that crop yields were subjected to different constraints (e.g., disease, pest, and drought) except for N, P, or K supply. In the radish database used for this study, the average value of HI was 0.64 kg kg^−1^, ranging from 0.34 to 0.91 kg kg^−1^. Only few plant samples had a HI < 0.4 and lot of plants with a HI within 0.4–0.8. The corresponding yield with lower HI (<0.4) was below 10 t ha^−1^ (fresh weight) and the data were excluded. However, the mean values of most parameters including yield, nutrient uptake, and IEs were not affected when excluded the data with a HI < 0.4 due to the large number of observations. Therefore, the HI value of 0.4 was chosen to be a threshold value to exclude data from the QUEFTS model calibration.

**Figure 2:**
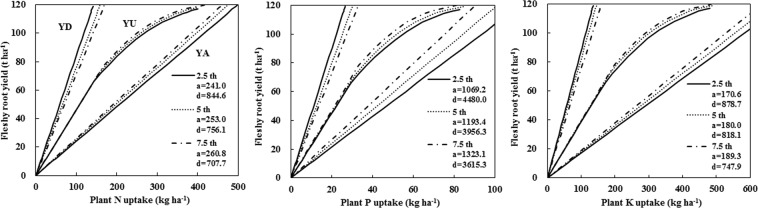
Radish fleshy root yield in relation to plant nutrient uptake at different (**a**,**d**) constants. The calculations excluded the upper and lower 2.5 (set I), 5 (set II), and 7.5 (set III) percentiles of all internal efficiency data (HI ≥ 0.4). YD, YA, and YU represent the maximum dilution, maximum accumulation, and balanced uptake of N, P, and K in plant dry matter, respectively. The potential yield was set at 120 t ha^−1^.

According to previous findings in grain crops, cassava, and sweet potato^14,22,34^, the borderlines of the maximum nutrient accumulation (a) and the maximum nutrient dilution (d) were the 2.5th and 97.5th percentiles of the calculated IE of a nutrient. From the sensitivity test of different datasets, we excluded the upper and lower 2.5th (set I), 5.0th (set II), and 7.5th (set III) percentiles of all IE data. The relationship between fleshy root yield and nutrient accumulation in total plant dry matter under a potential yield of 120 t ha^−1^ (i.e., maximum attainable yield) was estimated using the QUEFTS model. The nutrient requirements simulated by the QUEFTS model were similar for all three sets (Fig. 2), except at the target yields approaching the potential yield. Similar findings have been reported in the previous studies on grain crops^14,17,18^. Because of the large variability in the IEs among fields, we proposed that set I were used as a standard parameter set in the QUEFTS model to estimate the balanced nutrient uptake by radish and the relationship between fleshy root yield and nutrient accumulation. The a and d constants of set I were 241.0 and 844.6 kg kg^−1^ for N, 1069.2 and 4480.0 kg kg^−1^ for P, and 170.6 and 878.7 kg kg^−1^ for K, respectively (Fig. 2). In the present study, the calibration of the QUEFTS model was based on fresh matter basis but when it is converted to dry matter basis, the results of a and d constants were change only due to water content. The ratio of maximum dilution to maximum accumulation (d/a) for N (3.5) was less than for P (4.2) and K (5.1), indicating that a specific yield of radish relied on a relatively narrow range of N uptake, which is consistent with the results of an earlier study involving rice^14^. Therefore, precise N supply is more important for a stable yield formation relative to P and K, although both N, P, and K are essential to increase and maintain vegetable yield.

### Estimating N, P, and K requirements

**Figure 3:**
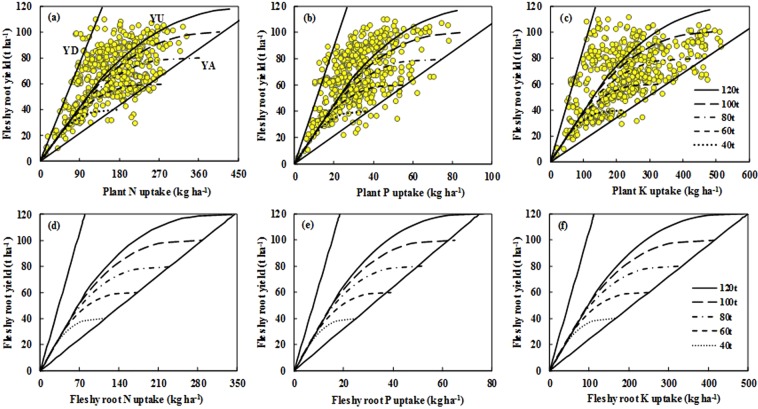
Relationships between fleshy root yield and N, P, and K accumulation in the total plant dry matter at maturity (**a**–**c**) and fleshy root N, P, and K removal in fleshy root dry matter (**d**–**f**) under different potential yields predicted by the QUEFTS model. YD, YA, and YU represent the maximum dilution, maximum accumulation, and balanced uptake of N, P, and K in the total plant dry matter or in the fleshy roots dry matter, respectively. These parameters were calculated by the QUEFTS model after excluding the upper and lower 2.5 percentiles of all internal efficiency data (HI ≥ 0.4). The potential yield ranged from 40 to 120 t ha^−1^.

The QUEFTS model calculated the N, P, and K requirements to achieve a certain target yield across potential yields ranging from 40 to 120 t ha^−1^ (Fig. 3a–c). The differences in the balanced requirements of N, P, and K (YU) simulated by the QUEFTS model for targeted fleshy root yields were large due to the different potential yields. However, the whole-plant N:P:K ratio required to produce 1000 kg fleshy roots was always the same in the linear part of the response curve regardless of the potential yield. The model predicted a linear increase in fleshy root yields if nutrients were taken up in balanced amounts until the yield reached about 60%–70% of the potential yield. Moreover, the linear part of the relationship was always 70%–80% of the whole yield range. Nutrient requirements increased and IEs reduced drastically from the linear level when target yields approached the potential yield (Table 4). These observations are consistent with those of earlier studies on grain crops^17–19^ and sweet potato^34^. That is more likely that IEs are greater at lower yields, where plant growth is mainly limited by nutrient availability. Therefore, it will be more profitable to farmers by balancing the application of NPK fertilizers to maximize nutrient use efficiency than to pursue higher yield targets closer to potential yield.

**Table 4:** Balanced nutrient uptake requirements, internal efficiencies (IE, kg fleshy root per kg nutrient), and the reciprocal internal efficiencies (RIE, kg nutrient per 1000 kg fleshy root) of N, P, and K simulated by the QUEFTS model to achieve specific radish fleshy root yield targets and a potential yield of 120 t ha^−1^.

Yield (kg ha^−1^)	Required nutrient uptake (kg ha^−1^)			Internal efficiency (kg kg^−1^)			Reciprocal internal efficiency (kg t^−1^)		
	N	P	K	N	P	K	N	P	K
0	0.0	0.0	0.0	0.0	0.0	0.0	0.0	0.0	0.0
6000	12.9	2.7	15.5	465.1	2222.2	387.1	2.15	0.45	2.58
12000	25.8	5.4	31.0	465.1	2222.2	387.1	2.15	0.45	2.58
18000	38.7	8.1	46.5	465.1	2222.2	387.1	2.15	0.45	2.58
24000	51.6	10.8	62.0	465.1	2222.2	387.1	2.15	0.45	2.58
30000	64.5	13.5	77.5	465.1	2222.2	387.1	2.15	0.45	2.58
36000	77.4	16.2	93.0	465.1	2222.2	387.1	2.15	0.45	2.58
42000	90.3	18.9	108.5	465.1	2222.2	387.1	2.15	0.45	2.58
48000	103.2	21.6	124.0	465.1	2222.2	387.1	2.15	0.45	2.58
54000	116.1	24.3	139.5	465.1	2222.2	387.1	2.15	0.45	2.58
60000	129.0	27.0	155.0	465.1	2222.2	387.1	2.15	0.45	2.58
66000	143.9	29.7	172.4	458.7	2222.2	382.8	2.18	0.45	2.61
72000	161.2	33.2	193.2	446.7	2168.7	372.7	2.24	0.46	2.68
78000	179.8	37.1	215.5	433.8	2102.4	361.9	2.31	0.48	2.76
84000	200.0	41.2	239.7	420.0	2038.8	350.4	2.38	0.49	2.85
90000	222.3	45.8	266.5	404.9	1965.1	337.7	2.47	0.51	2.96
96000	247.5	51.0	296.6	387.9	1882.4	323.7	2.58	0.53	3.09
102000	276.7	57.0	331.7	368.6	1789.5	307.5	2.71	0.56	3.25
108000	312.7	64.4	374.7	345.4	1677.0	288.2	2.90	0.60	3.47
114000	362.4	74.7	434.4	314.6	1526.1	262.4	3.18	0.66	3.81
120000	441.9	86.9	490.3	271.6	1380.9	244.7	3.68	0.72	4.09

The balanced nutrient requirement of 2.15 kg N, 0.45 kg P, and 2.58 kg K predicted by the QUEFTS model was required to produce 1000 kg fleshy roots, and corresponding IE values for N, P, and K were 465.1, 2222.2, and 387.1 kg kg^−1^ (Table 4). For a comparison, the average values of IE-N, IE-P, and IE-K from field experiments in the radish database were 455.0, 2327.3, and 361.8 kg kg^−1^. These differences were probably due to nutrient imbalance application by farmers from field experiments in the radish database and the diversity of potential yields at the different experimental sites. The challenge for future will be to improve the IEs by applying balanced N, P, and K nutrients and optimizing agronomic management at constant or increased fleshy root yield levels and nutrient use efficiencies. The RIEs simulated by the QUEFTS model (i.e., 2.15 kg N t^−1^, 0.45 kg P t^−1^, and 2.58 kg K t^−1^) were only for the linear portion of the predicted balanced nutrient uptake curve, so less than the corresponding values obtained from field experiments in the radish database (i.e., 2.45 kg N t^−1^, 0.49 kg P t^−1^, and 3.41 kg K t^−1^). It was confirmed by the observed increase as the target yield increased above 60%–70% of the potential yield^35^. The ratio of NPK uptake simulated by the QUEFTS model in the linear part was 4.78:1:5.74, while the ratio of the average of measured NPK uptake from field experiments in the radish database was 5.00:1:7.05. There was a difference between the K uptake predicted by the QUEFTS model and the average of measured nutrient uptake from field experiments in the radish database. This discrepancy was due to the nutrient uptake estimated by the QUEFTS model was based on the optimal nutrient requirements^15,16^. However, more K uptake values in this study from field experiments in radish database were concentrated near the maximum nutrient accumulation boundary and only a few datasets revealed a deficiency, indicating the excessive K uptake by radish (Fig. 3c), and reflect the high soil K content and excessive application of K fertilizer. Some N and P uptake data were not close to the optimal nutrient uptake line as predicted by the QUEFTS model (Fig. 3a,b), indicating that N and P accumulation in radish plant showed both deficient and excessive in the main radish-producing regions, and reflect the imbalanced application of N and P fertilizers. This was mainly due to the fact that the N, P, and K fertilizers were not applied according to the indigenous supply of nutrient in soil or the need of plant in many nutrient management practices or treatments including farmers’ practices, nutrient omission treatments, and CK (check no fertilizer application), etc, from field experiments in the radish database.

The QUEFTS model simulated the fleshy root nutrient removal, which may be useful for the rational fertilization where P and K removed in the fleshy root should be returned to the soil through fertilization to avoid soil nutrient depletion. The a and d constants for the fleshy root nutrient removal were calculated through eliminated the upper and lower 2.5 percentiles of all fleshy root nutrient IE values (kg fleshy root per kg nutrient removed in fleshy root dry matter) (HI ≥ 0.4). Results indicated that the curve of balanced fleshy root nutrient removal was similar to the balanced nutrient requirements for the total plant under different potential yields (40–120 t ha^−1^) (Fig. 3d–f). The QUEFTS model analysis showed that the balanced N, P, and K removal by fleshy root to produce 1000 kg fleshy root in the linear part of the curve were 1.34, 0.30, and 1.93 kg, respectively, regardless of the potential yields, and the N:P:K ratio in the fleshy root was 4.47:1:6.43. Compared with the balanced nutrient uptake in total plant, about 62%, 67%, and 75% of the N, P, and K accumulated in the fleshy root and were removed from the soil. These values should be useful for fertilizer recommendations to maintain soil fertility.

### Validation of the QUEFTS model

**Figure 4:**
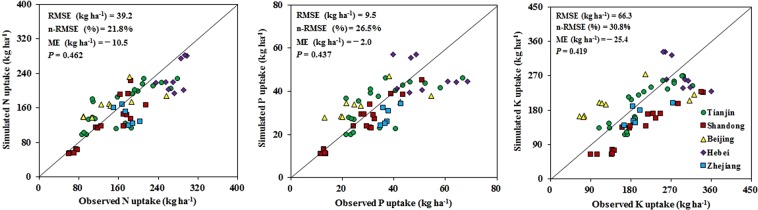
Comparisons of the simulated and observed data of N, P, and K uptake in whole radish plant dry matter in 2016–2018. The observed data were obtained from field experimental plots where the Nutrient Expert (NE) decision support system was applied. The simulated nutrient uptake data were estimated using the QUEFTS model.

We analyzed the observed and simulated nutrient uptake data for multiple field experiments in Tianjin, Shandong, Beijing, Hebei, and Zhejiang in 2016–2018 to validate the QUEFTS model. The RMSE, nRMSE, and ME were used to evaluate the QUEFTS model. The RMSE values of N, P, and K were 39.2, 9.5, and 66.3 kg ha^−1^, respectively, while the nRMSE values of N, P, and K were 0.22, 0.27, and 0.31, respectively. Moreover, the ME values of N, P, and K were −10.5, −2.0, and −25.4 kg ha^−1^, respectively (Fig. 4). These results implied that the simulated N and P uptake was consistent with the actual nutrient uptake, while there were differences for the K uptake. However, across all experiments, the observed N, P, and K uptake in total plant dry matter was distributed near the 1:1 line, and the *P* values for N, P, and K were 0.462, 0.437, and 0.419, respectively. These observations indicated that the observed and simulated nutrient uptake values were similar, with no significant deviations. The experimental results confirmed that the QUEFTS model could be used to predict nutrient requirement and to develop fertilizer recommendations.

## Conclusions

Datasets involving radish yield and nutrient uptake were built for analyzing the relationship between them, and to evaluate the optimal nutrient requirements using the QUEFTS model. Regardless of yield potential, the model predicted a linear increase in fleshy root yields if nutrients were taken up in balanced amounts of 2.15 kg N, 0.45 kg P, and 2.58 kg K per 1000 kg of fleshy root until the yield reached about 60%–70% of the potential yield. The N, P, and K nutrient removal by fleshy root was also simulated by QUEFTS model for the development of fertilizer recommendations. Field validation demonstrated that the QUEFTS model can be used as a practical and robust tool for estimating nutrient requirements of radish. Therefore, the QUEFTS model can help develop valuable tools (e.g., Nutrient Expert system) with more feasible and site-specific nutrient management strategies for radish.

## Data Availability

The datasets generated and/or analyzed during this study are available from the corresponding author upon reasonable request.
